# The Role of Stream Water Carbon Dynamics and Export in the Carbon Balance of a Tropical Seasonal Rainforest, Southwest China

**DOI:** 10.1371/journal.pone.0056646

**Published:** 2013-02-20

**Authors:** Wen-Jun Zhou, Yi-Ping Zhang, Douglas A. Schaefer, Li-Qing Sha, Yun Deng, Xiao-Bao Deng, Kai-Jie Dai

**Affiliations:** 1 Key Laboratory of Tropical Forest Ecology, Xishuangbanna Tropical Botanical Garden, Chinese Academy of Sciences, Mengla, Yunnan, China; 2 Xishuangbanna Tropical Botanical Garden, Chinese Academy of Sciences, Kunming, China; 3 Xishuangbanna Station for Tropical Rain Forest Ecosystem Studies, Chinese Ecosystem Research Net, Mengla, Yunnan, China; 4 University of Chinese Academy of Sciences, Beijing, China; University of Delaware, United States of America

## Abstract

A two-year study (2009 ∼ 2010) was carried out to investigate the dynamics of different carbon (C) forms, and the role of stream export in the C balance of a 23.4-ha headwater catchment in a tropical seasonal rainforest at Xishuangbanna (XSBN), southwest China. The seasonal volumetric weighted mean (VWM) concentrations of total inorganic C (TIC) and dissolved inorganic C (DIC) were higher, and particulate inorganic C (PIC) and organic C (POC) were lower, in the dry season than the rainy season, while the VWM concentrations of total organic C (TOC) and dissolved organic C (DOC) were similar between seasons. With increased monthly stream discharge and stream water temperature (SWT), only TIC and DIC concentrations decreased significantly. The most important C form in stream export was DIC, accounting for 51.8% of the total C (TC) export; DOC, POC, and PIC accounted for 21.8%, 14.9%, and 11.5% of the TC export, respectively. Dynamics of C flux were closely related to stream discharge, with the greatest export during the rainy season. C export in the headwater stream was 47.1 kg C ha^−1^ yr^−1^, about 2.85% of the annual net ecosystem exchange. This finding indicates that stream export represented a minor contribution to the C balance in this tropical seasonal rainforest.

## Introduction

Streams and small inland rivers are important links between terrestrial and aquatic ecosystems. Cole et al. [1] suggested that inland waters export 1.9 Pg C yr^−1^, indicating that regional carbon (C) balances can influence transport into large terrestrial rivers and oceans [2]. Recently, several studies have focused on dissolved organic C (DOC), dissolved inorganic C (DIC), particulate inorganic (PIC) and organic C (POC), and even gaseous C (CO_2_, CH_4_) in catchment runoff, and on their role in C exports from ecosystems [2]–[8].

Previous studies showed that the export of dissolved and gaseous C with rivers and streams may vary among forest ecosystems. Shibata et al. [9] found that sum of DIC and DOC export by stream water (7.6 g C m^−2^ yr^−1^) accounted for only 2% of net ecosystem exchange (NEE) in cool temperate forests of northern Japan, whereas in Canadian boreal forests, C export from surface waters accounted for NEE from 9.5% to 16.4% [10]. In the Amazon, Richey et al. [6] demonstrated that outgassing of CO_2_ (1.2±0.3 Mg ha^−1^ yr^−1^) from rivers and wetlands constituted an important C loss. Also Lloret et al. [8] demonstrated the key role of streams in the C balance of forest catchments in the Amazon Basin. Neu et al. [2] showed that C transported by water comprised about 20% of the total annual C exchange across tropical forest canopies. The roles of surface water in C export vary because of diversity in geographic location, basin-specific soil and vegetation types, catchment topography, climate, and upland-wetland flow paths in forests [4], [7], [11]–[16]. As a result, by ignoring the export of CO_2_, DOC, DIC, PIC and POC via hydrological pathways, terrestrial C budgets are incomplete and net C sequestration could be overestimated [1].

**Figure 1:**
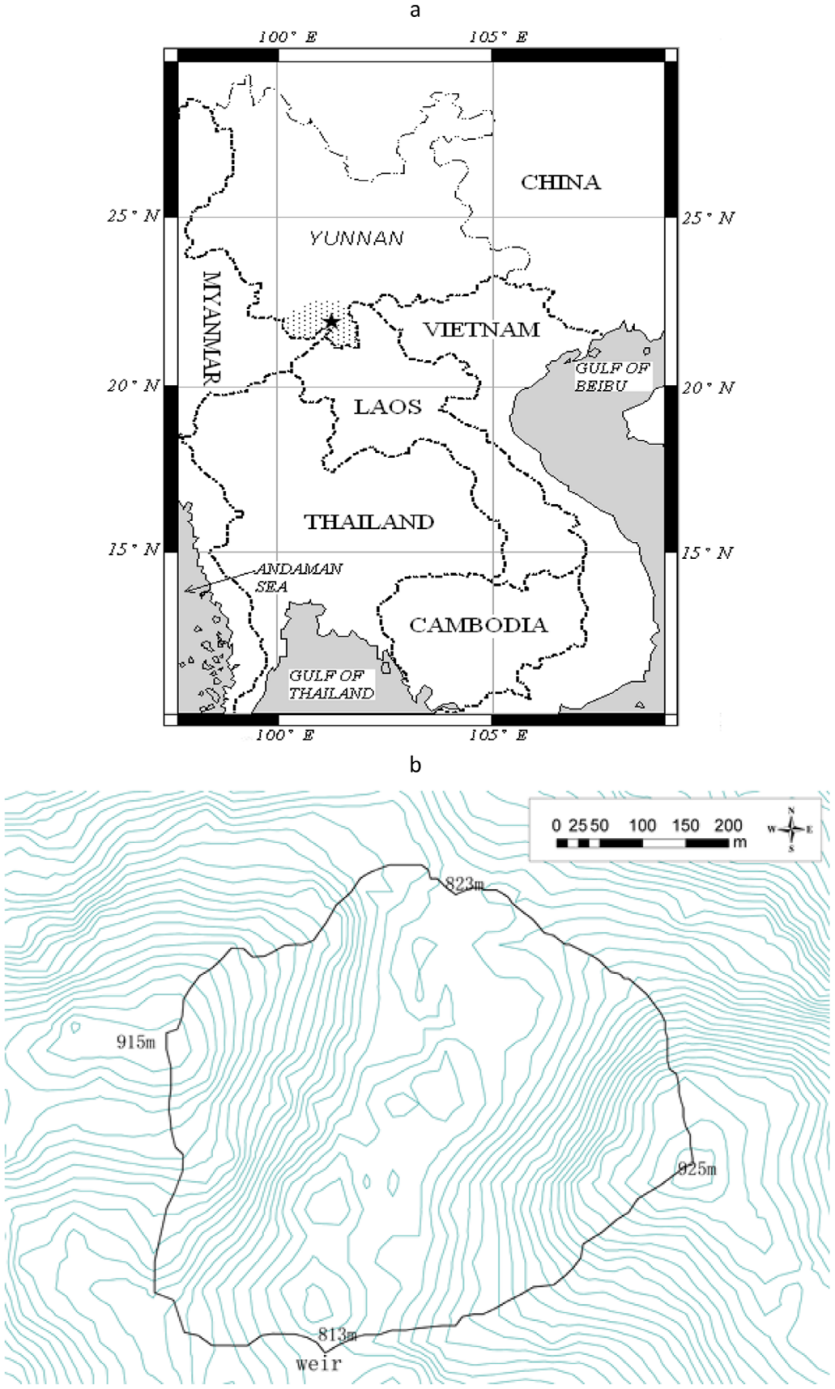
Study site description in Xishuangbanna tropical seasonal rainforest, Southwest China. (a) Location of the study area (indicated by the black star). (b)The catchment site description was from the Advanced Spaceborne Thermal Emission and Reflection Radiometer (ASTER) Global Digital Elevation Model (GDEM) that is a product of METI and NASA.

Surface water and wetland play substantial roles in C balance in the Amazon, the largest tropical forest region in the world [2], [6], [8]. So far, little is known about the importance of C export by headwater streams on the carbon balance of tropical seasonal rain forests (TSRF) at the northern edge of the tropical zone in southwest China (Figure 1). Despite its relatively high latitude, tropical seasonal rain forest has a moist tropical climate due to the influence of the Himalayas. It is unique in terms of forest type, differing from those in the equatorial region of Southeast Asia and has highly diverse and mixed types of floristic compositions due to its unique geographical location between a tropical zone to the south and a subtropical zone to the north [17]. Consequently, the tropical seasonal rain forest in southwest China is an important biogeographic area in Southeast Asia. Tan et al. [18] and Zhang et al. [19] have reported that TSRF in Xishuangbanna (XSBN) is a small net C source. Accounting for TC export with stream water may make the loss of C from TSRF at XSBN even larger than earlier anticipated. In order to clarify the role of C export by headwater streams, a study was therefore undertaken in TSRF at XSBN. The objectives of this study were (1) to ascertain the seasonal dynamics of different C components (DIC, DOC, PIC, POC, TIC, TOC, and TC), and (2) to assess the contribution of stream export to the C balance in this tropical seasonal rainforest ecosystem.

## Materials and Methods

### Ethics Statement

All necessary permits were obtained from Xishuangbanna National Nature Reserve for the described field studies which did not involve endangered or protected species.

### Study Site

The study area in XSBN (Dai autonomous prefecture), Yunnan province, China (21.16° N, 101.04° E) (Figure 1), is influenced by the Southwest monsoon and dominated by North Tropical Monsoon weather, with annual average temperature 21.5°C, annual average rainfall 1557 mm and average relative humidity 86%. Based on precipitation data, the rainy season (with 84.1% of the total annual precipitation) [20], [21], is between May and October. The dry season is between November and April.

The experimental site is located in the centre of the National Forest Reserve in Menglun, Mengla County, Yunnan province, with relatively little human disturbance. The dominant trees are *Terminalia myriocarpa* and *Pometia tomentosa*, which is typical of tropical forest [17]. The total catchment area is 23.4 ha, the slope is 12°∼18°, and the soil type is oxisol formed from Cretaceous yellow sandstone with a pH value of 4.5∼5.5 and a clay content of 19.5%∼29.5% [22].

### Experimental Set-up

#### Hydrological observations

At the watershed outlet, a 90° V-notch weir instrumented with a water-level recorder was installed. The recorder was set to take averaged discharge measurements at 5-min intervals. Daily and monthly discharges were calculated separately from the stream-height data, as follows:
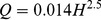
(1)

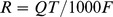
(2)


Where Q = discharge (m^3^/s); H = water head (m); R = runoff (mm); T = time (s); and F = catchment area (km^2^). Stream water temperature (SWT) was recorded at the mid-point of stream depth near the stream outlet. Measurements were made every half hour and stored in a data logger.

#### Water sample sampling and analysis

Stream water was sampled in the middle of the stream outlet. Stream water samples were collected between 8∶00 and 9∶00 am local time at the sampling site in high-density polyethylene (HDPE) bottles; sampling bottles were completely filled, allowing no headspace. Bottles were rinsed with distilled water after being washed with 3% HCl solution. Bottles were pre-rinsed three times with the stream water before sample collection. The study was done during two full calendar years, from 1.1.2009 to 31.12.2010. During the dry season, stream water samples were collected once per week, in addition to daily samples during three consecutive days following rain events. Stream water was sampled twice per week during the rainy season in 2009, and once per week in 2010. All water samples were immediately transported to the laboratory in insulated bags.

Following the analysis method of Baker et al. [23], all samples were vacuum-filtered through 0.45-µm GFF (Tianjinshi Dongfang Changtai Environmental Protection Technology Co. Ltd., China) pre-rinsed with deionized water and sample water under vacuum. The filtered and unfiltered water samples were analysed for DOC/DIC and TOC/TIC by TOC/TN analyser (LiquiTOC II, Elementar Analyses System GmbH, Germany) respectively, within 24 hours.

The TOC/TN analyser allows particle size up to 200µm. So in this study, the diameter of particulate matter (PIC and POC) was defined from 0.45 µm to 200 µm. TIC and TOC were defined as less than 200 µm for all the water samples. Concentrations of PIC and POC were calculated by subtracting the DIC and DOC concentrations from the TIC and TOC concentrations, respectively.

### Calculations and Statistics

The monthly volume-weighted mean (VWM) concentrations were computed as follows:
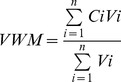
(3)


Where VWM is the volume-weighted mean concentration, Ci is the concentration (mg L^−1^), and Vi is the runoff (m^3^ s^−1^) at sampling time.

The monthly C flux was calculated as the monthly VWM C concentration multiplied by monthly discharge. We calculated TIC, DIC, TOC, and DOC flux directly, and calculated PIC, POC, and TC flux as follows:
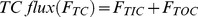
(4)

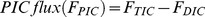
(5)

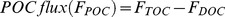
(6)


Where F indicates flux.

The correlations among stream discharge, stream water temperature on the one hand, and concentrations of TIC, TOC, DIC, DOC, PIC, and POC on the other, as well as the correlations among different carbon components, were tested using the Pearson correlation (two tailed), employing the software SPSS 15.0.

## Results

### Seasonal Variations of Rainfall, Stream Runoff, and Stream Water Temperature

**Figure 2:**
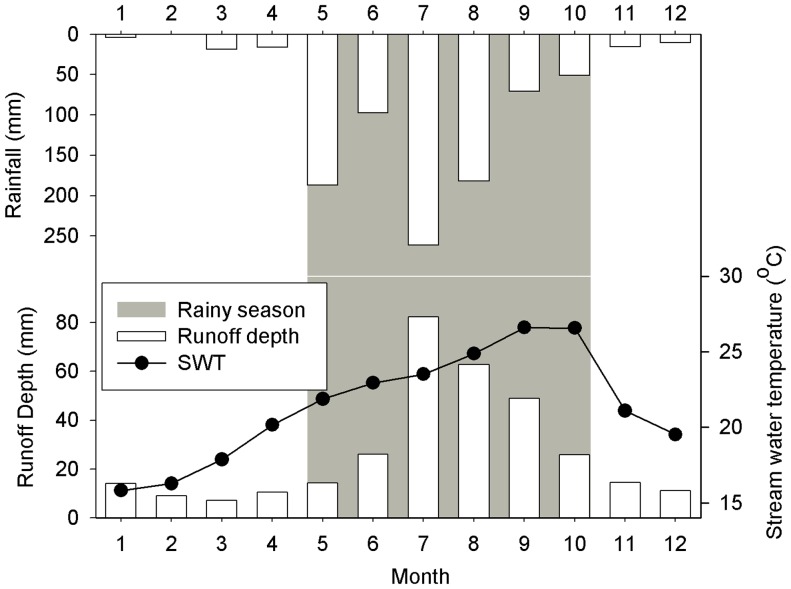
Annual dynamics of rainfall, runoff depth, and stream water temperature in the seasonal tropical rainforest. Values are averages for 2009 and 2010.

The average annual rainfall and runoff for the two years were 1026.1 mm and 326.9 mm, respectively. These values are less than the past 40 years means [24]. Rainfall and runoff were higher during the rainy season (average 848.9 mm and 279.1 mm, respectively) than during the dry season, confirming earlier reports on the seasonal dynamics of rainfall and stream discharge [20], [25].The seasonal dynamics of rainfall and runoff showed similar patterns and were well correlated (r = 0.794, p<0.001, n = 24; Figures 2, S1).

Average SWT was 21.4°C, with lowest values in January (15.8°C) and peak values in September (26.6°C; Figures 2b, S1).

### C concentration Dynamics

**Figure 3:**
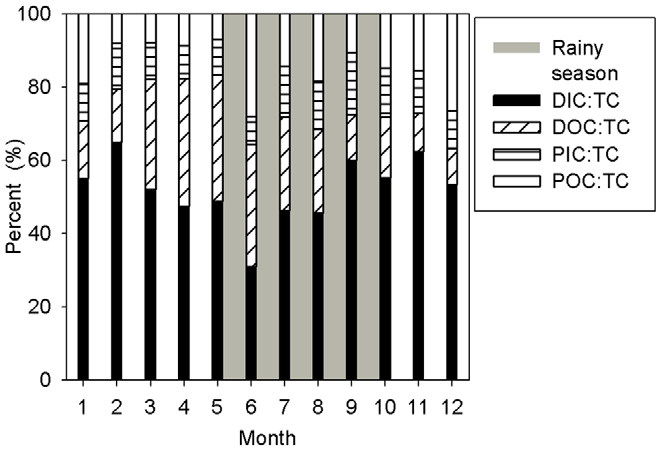
Annual dynamics of percentage difference of various carbon components to TC export, in the headwater stream.

DIC was the largest component of TC. The rank order of the overall contribution of different C forms to TC was as follows: DIC (51.8%)>DOC (21.8%) >POC (14.9%) >PIC (11.5%) (Figure 3). However, these contributions differed seasonally: the contribution of DIC to TC was lowest in July (31.0%) and highest in February (64.9%), DOC had the highest contribution to TC in April and May (35.0%) and the lowest in December (10.0%), and the contribution of POC to TC was greatest in July (Figure 3). The DIC: DOC ratio and its monthly variation (2.9 and 67.1%, respectively) were higher than those for PIC: POC (0.8 and 47.3%, respectively).

Seasonal variations in VWM concentration were different among the various C components (Table 1). The VWM concentrations of TIC and DIC were higher during the dry season than during the rainy season, others were similar.

**Table 1:** Concentration and export of various carbon components in a headwater stream in the tropical seasonal rainforest of Xishuangbanna, Southwest China.

	Carbon concentration (mg C L^−1^)			Carbon export (kg C ha^−1^)				
Season	Dry season	Rainy season	Annual	Dry season	Rainy season	Annual average	2009	2010
TIC	10.1	6.9	7.5	7.6	21.1	28.7	32.5	24.9
TOC	6.6	6.6	6.6	3.9	14.5	18.4	21.2	15.7
DIC	8.9	5.4	6.0	6.4	16.6	22.9	26.0	19.9
DOC	3.7	3.7	3.7	2.2	8.6	10.8	13.4	8.1
PIC	1.6	1.7	1.7	1.2	4.6	5.8	6.5	5.0
POC	3.1	3.3	3.2	1.7	5.9	7.6	7.7	7.5
TC				11.5	35.6	47.1	53.5	40.6

Carbon concentration is the seasonal volumetric weighted mean (VWM) concentration of 2009 and 2010.

**Figure 4:**
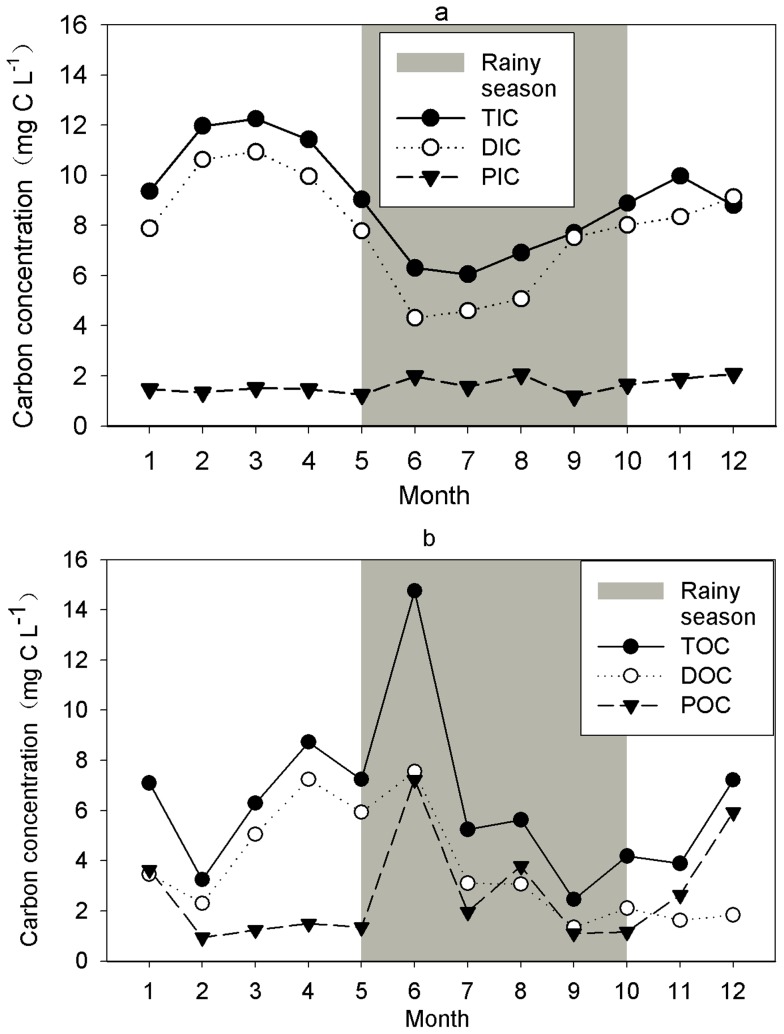
Annual dynamics of monthly volumetric weighted mean concentrations of carbon components in seasonal tropical rainforest. (a) Annual dynamics of monthly volumetric weighted mean concentrations of TIC, DIC, and PIC. (b) Annual dynamics of monthly volumetric weighted mean concentrations of TOC, DOC, and POC.

Monthly VWM concentrations of TIC and DIC were significantly correlated (r = 0.956, p<0.001, n = 24). The highest monthly VWM concentration was in March as discharge was the lowest; the lowest concentrations were in June and July while stream discharges were relatively high (Figures 2b, 4a). Both TIC and DIC were negatively correlated to discharge and SWT (Table 2). The highest and lowest monthly VWM concentrations of PIC occurred in August (2.0 mg L^−1^) and September (1.2 mg L^−1^) while discharge was high. Although floods increased DOC and POC concentrations, the highest VWM concentrations of TOC, DOC and POC occurred during the beginning of the rainy season (Figure 4b) at intermediate values of discharge (Figure 2b). The lowest values of TOC and DOC were in September during relatively high discharge, but POC was lowest in February when discharge was low. The seasonal dynamic of DOC was different from DIC (r = 0.157, p = 0.464, n = 24), but those of PIC and POC were similar (r = 0.515, p = 0.010, n = 24). The annual variation of PIC (coefficient of variation (CV) = 19.2%) was less than that of TIC (CV = 23.1%) and DIC (CV = 28.4%). The rank order of the coefficients of variation of organic C forms was as follows: POC (CV = 76.4%), DOC (CV = 58.9%), TOC (CV = 51.2%).

**Table 2:** Pearson correlations for monthly average stream discharge, monthly average SWT, and monthly VWM concentration of carbon components in 2009 and 2010 in a headwater stream in the tropical seasonal rainforest in Xishuangbanna, Southwest China.

Parameters	TIC	TOC	DIC	DOC	PIC	POC
Monthly Q (n = 24)	−0.658**	−0.140	−0.661**	−0.139	0.057	−0.079
SWT (n = 24)	−0.520**	−0.166	−0.482*	−0.103	−0.150	−0.186

* Correlation is significant at the 0.05 level (2-tailed);

** Correlation is significant at the 0.01 level (2-tailed).

### Stream C Flux Dynamics and Distribution

**Figure 5:**
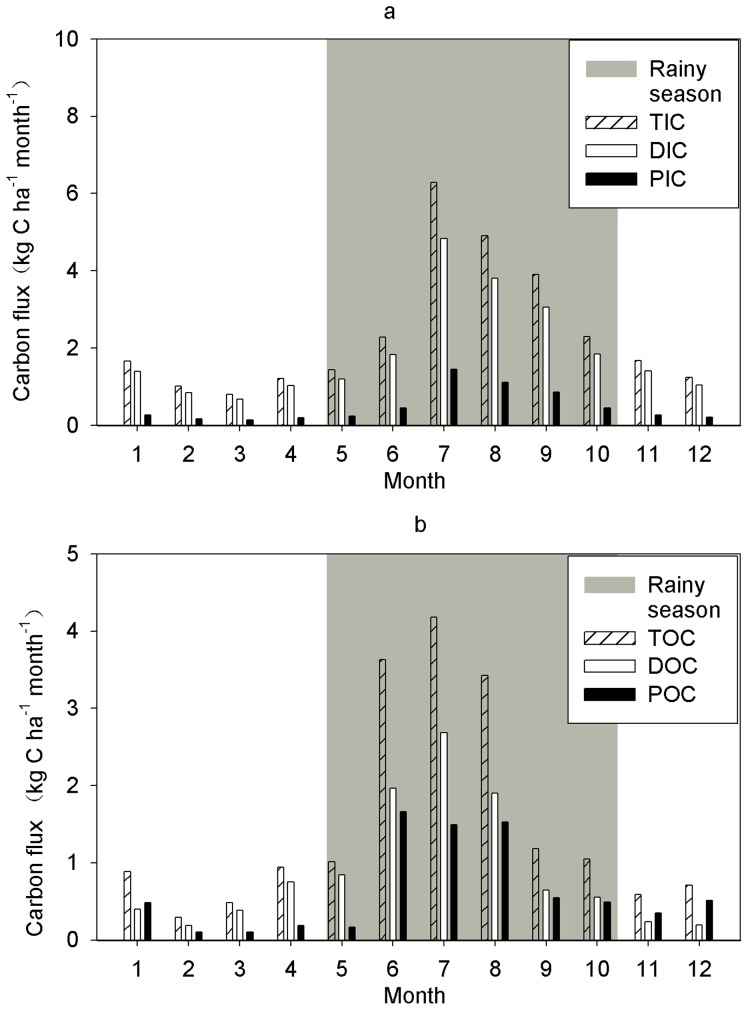
Annual dynamics of flux of carbon components in a headwater stream in tropical seasonal rainforest. (a) Annual dynamics in flux of TIC, DIC, and PIC. (b) Annual dynamics in flux of TOC, DOC, and POC.

Annual TC export was 53.9 kg ha^−1^ and 40.7 kg ha^−1^ in 2009 and 2010, respectively. The dynamics of the fluxes of C differed between the various compounds. With the exception of POC, the greatest flux of all C components occurred in July when discharge was highest (Figures 2, 5). By contrast, POC export was greatest in June when discharge was intermediate. Due to low discharge also C flux was low in February (DOC, TOC) and in March (inorganic C and POC). Measures of seasonal C export (Table 1) showed that most of the C export for all components occurred during the rainy season.

### Contribution Stream Water C Flux in the C Balance of Tropical Seasonal Rainforest

**Figure 6:**
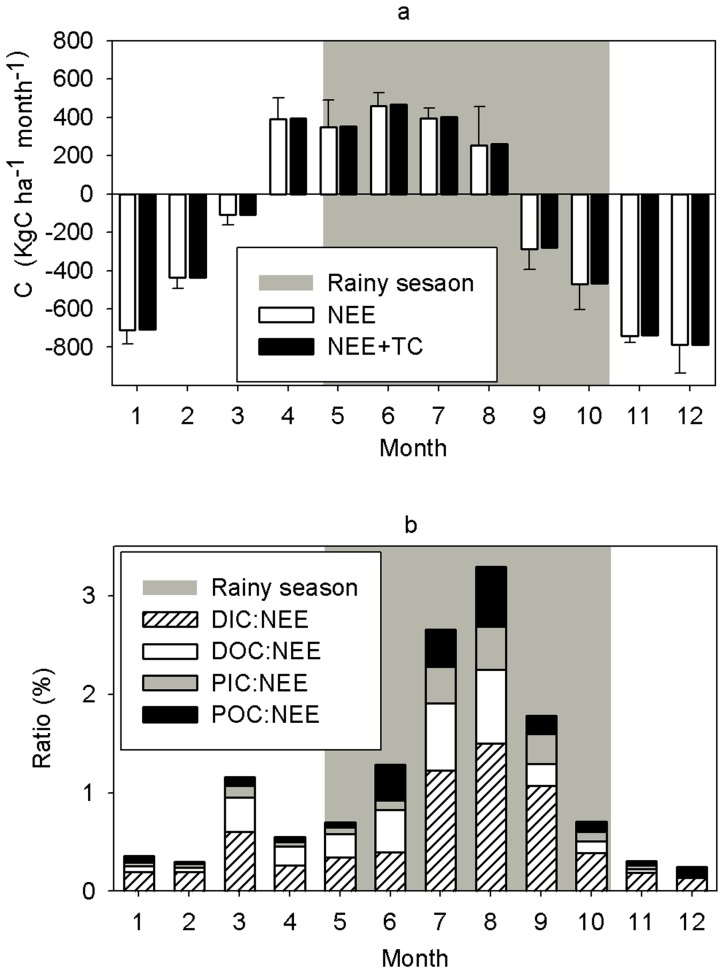
Annual dynamics of ratio of stream carbon export to net ecosystem CO_2_ exchange (NEE). (a) Annual dynamics of NEE [19] and the sum of NEE and TC exported by headwater stream. (b) Annual dynamics of ratios of annual flux of DIC (DIC: NEE), DOC (DOC: NEE), PIC (PIC: NEE), and POC (POC: NEE) to NEE.

Based on seasonal NEE dynamics (Figure 6a) [19], TC export resulted in an increased net C export ratio (TC: NEE) from 0.54% to 3.30% from April to August, and in a decrease from September to March, ranging from 0.20% to 1.80%. In total, stream C export represented a mean 2.85% (2009, 3.25%; 2010, 2.45%) increase in the annual net carbon export (1660 kg C ha^−1^ yr^−1^) [19] in the TSRF. The ratios of DIC, DOC, PIC, and POC export to NEE showed the highest fractions of the C components’ flux to NEE occurred in August (DIC: NEE = 1.50%, DOC: NEE = 0.75%, PIC: NEE = 0.44%, POC: NEE = 0.60%). The lowest absolute value of ratios of DIC, DOC, and PIC to NEE were observed in November (DIC: NEE = 0.13%, DOC: NEE = 0.024%, PIC: NEE = 0.025%), whereas the lowest value of POC: NEE occurred in February (0.024%; Figure 6b).

## Discussion

### C Dynamics in a Headwater Stream in the Tropical Seasonal Rainforest of Xishuangbanna

Headwater stream C originates from surface soil, ground water, vegetation (dead and alive), roots and microbial biomass in the forest [8], [26], [27]. Stream C dynamics depended on rainfall and discharge dynamics in this study and other small catchments [13], [16], [28]. Accurate calculation of C export with stream water requires representative sampling [12], [16]. Our sampling campaigns were throughout 2 years, and included both base flow and storm flow conditions (Figure S1), so that carbon components in stream water were sampled across a wide range of discharge rates (Figure S2). Otherwise, our sampling revealed little particle matter larger than 200 µm only in March when discharge was relatively low (Figure 1). Therefore, this approach ensures the relatively high accuracy of C export calculations, which includes all C matter less than 200 µm in size. Accordingly, our calculations of TC and PC export excluded coarse C particles (>200 µm) in this study.

DIC was the most important C component (Figure 4, Table 1) of C export in the headwater stream of TSRF. This is consistent with the observation that the mineral soil has little organic C (23.88 g kg^−1^) [22] and that there are few storm events (Figure S1) [2], [8], which tend to be higher in DIC than in DOC. Ground water flow, which dominates base flow, is a continuous C conduit from landscape to stream in Amazonian [2], [7], [8], [29], [30] and British forests [23]. In streams, groundwater-derived DIC is significantly diluted by surface runoff and interflow [24] and by PIC transferred to the stream by surface runoff [31], [32] and lateral movement of soil water during rain events [8], [23]. In addition, TIC and DIC concentrations in stream water may decrease with increases in SWT (Table 2) during the rainy season, due to its microbial transformation to DOC or gaseous C [13], [31], [32]. Similar observations were reported for small streams in the Amazon [32] and in northern California [31].

DOC was the dominated organic carbon form in the headwater stream (Figure 5, Table 1). TOC and DOC dynamics were similar (Figures 4b, S2, Table 1). The annual variance of DOC (CV = 58.9%) concentration was more than that of DIC (CV = 28.4%), and correlations between DOC and stream water discharge differ from those for DIC (Figures 2, S2, Table 2) suggesting different sources for DOC and DIC. Litter fall peaks in late March [33], [34], releasing dissolved organic matter (DOM) through decomposition [7]. Lower stream discharge in March and April further increased the DOM concentration. At the beginning of the rainy season, organic C in surface soil and litter leachate is transported to the headwater stream by surface runoff, interflow water, and canopy throughfall [26], [29], [35] due to persistent rain events [20]. In addition, a large share of DOC from soil is “flushed” during the last rainstorms of the dry season and the first storms in the rainy season [36]. Also, stream DOC concentration peaked in June (Figures 4b, S2), and was lowest in September (Figure 4b) when litter had been decomposed and the store of DOC in the soil had leached gradually during the rainy season [36] (Figure S2). This explains the weak relationship between discharge and DOC concentration (Table 2), but disagrees with the strong positive or negative correlations of streams having varying agricultural land-use intensities in their catchments [28]. The VWM concentrations were similar in the rainy season and the dry season, which contrasts other studies showing DOC concentrations higher during the rainy season [8], [16], [32].

Stream C export increased as stream discharge increased (Figures 2b, 4). This result supports the notion that organic and inorganic C export in watersheds is always dominated by runoff amount despite sometimes even smaller concentrations in stream water [3], [27]. TOC flux in this study (18.4 kg C ha^−1^ yr^−1^) was much less than that in Amazonian rivers and streams (100 kg ha^−1^ yr^−1^) [3], reflecting higher rainfall and stream discharge in the Amazon tropical region [3], [20]. In addition to stream discharge, C export is influenced by vegetation, soil type, and soil inorganic and organic carbon content [3], [8], [26], [28]. DIC flux was higher in this study than in the Southern Amazon (11.3 kg C ha^−1^ yr^−1^) [2], and was within the range of fluxes reported for Basse-Terre Island catchments, Lesser Antilles, during periods of low flow (1.7±0.9 to 14.8±9.4 kg C ha^−1^ yr^−1^) and flood (7.3±4.2 to 75.7±36.9 kg C ha^−1^ yr^−1^) [8] where runoff were higher than XSBN. TOC flux in XSBN was higher than that in subtropical forest in China (7.2 to 9.3 kg C ha^−1^ yr^−1^) [37], although the subtropical forest catchment had more soil carbon stock (164 t C ha^−1^) [38] than our site (87.0 t C ha^−1^) [39]. DOC export (12.0, and 9.5 kg C ha^−1^ yr^−1^ in 2010 and 2009, respectively) at XSBN was less than that measured in Juruena headwater catchments in Brazil (31.5 kg C ha^−1^ yr^−1^) [7], tropical volcanic islands in Guadeloupe (16.0±9.0 to 57.0±26.0 kg C ha^−1^ yr^−1^). Also, primary tropical forest (20.7±1.89 kg C ha^−1^ yr^−1^), secondary forest (18.9±1.4 kg C ha^−1^ yr^−1^ ), pine reforestation (17.9±9.0 kg C ha^−1^ yr^−1^ ) and cabbage cultivation (14.8±1.0 kg C ha^−1^ yr^−1^) in tropical highlands in northern Thailand [40], and a Wisconsin stream in a peat land catchment (25.0 kg ha^−1^ yr^−1^) [41] had higher C export rates than XSBN. The differences in C dynamics among these regions may reflect the soil type, stream chemistry, vegetation, or hydrology. Therefore, future studies should consider the complex mechanisms that underlie regional differences in C dynamics.

### Role of Stream Water Export in the C Balance

The contributions of the fluxes of all C components to the net C loss (as determined by the NEE) were determined (Figures 5, 6). Stream export increased C output from April to August but the NEE indicated that TSRF was C source in this period. From September to March carbon accumulation of TSRF was smaller than indicated by the C sink suggested by the NEE, due to organic C export with stream water (Figure 6a). In comparison with NEE, all stream water C components were small (Figure 6b; Table 3), even for TC. TC export in TSRF in XSBN (Table 3) matched or exceeded C emission due to stem respiration [42]. Compared to C emission due to soil respiration (SR) [43], and C sinks represented by litter fall [44], and fine root biomass production [45], the contribution of stream export was even smaller (Table 3). So, stream C export by headwater stream is negligible in the overall C balance (NEE, SR, litterfall, and root biomass production) of TSRF at XSBN.

**Table 3:** Ratios of total carbon export to different components of the carbon cycle in a tropical seasonal rainforest stream in Xishuangbanna, Southwest China.

Links of carbon cycle	kg C ha^−1^ yr^−1^	TC Ratio%	Reference
NEE	1660	2.85	[19]
Soil surface efflux	14564	0.32	[43]
Soil respiration	9491	0.50	
Litter respiration	3245	1.45	
Stem respiration	14∼47	100.31∼332.39	[42]
Litterfall mass	7180∼12850	0.37∼0.66	[44]
Fine root mass of 0–20 cm depth	6124	0.77	[45]
Living fine root mass of 0–20 cm depth	5418	0.87	
Dead fine root mass of 0–20 cm depth	707	6.66	

The TC ratio (%) indicates the ratio of TC export by stream to the amount of carbon in different components of the carbon cycle.

Our study contrasts many others, who have suggested that surface waters are an important export pathway for C in tropical regions [2], [6], [8] and boreal forests [10], [46]. Studies in the southern Amazon state of Mato Grosso showed TC (sum of DIC, DOC and fine-particle carbon) export by stream was 7.34% of NEE (1.5 Mg C ha^−1^ yr^−1^) [2], which is higher than the ratio of TC: NEE 2.85% (Table 3) in this study. In contrast, Shibata et al. [9] found that DIC and DOC output by stream water (7.6 g C m^−2^ yr^−1^) accounted for only 2% of NEE in cool-temperate forests of northern Japan, which is less than this study. Reason for relatively small C export by TSRF in the headwater stream at XSBN is that stream discharge was less than that of Amazon tropical regions [2], [6], [8], although NEE [19] and litter input [34] were similar in Amazon regions and XSBN. Furthermore, DOC export to the ecosystem C balance is small if adsorption to the soil matrix is strong [47], [48]. Clays particular oxides have greater potential to adsorb DOC compared with the clay-poor sandy podzolic soils found in Amazonian forest [26]. Hence, fluvial export of C from XSBN’s clay rich soils is likely to be lower in the present study area.

Based on the discussions above, the sources of different C components varied, leading to differences in the relative influence of stream discharge and SWT on C concentration and seasonal patterns. The relationship between stream discharge and C concentrations, and the distribution of C fractions differed in their influence on the C budget. A comparison of TC: NEE values in tropical seasonal rainforest at Xishuangbanna and in Amazon tropical forest and boreal forest indicates that stream export represents only a small component of the overall forest C balance in tropical seasonal rainforest.

## Supporting Information

Figure S1 **Sampling date of stream water 2009–2010 in tropical seasonal rainforest at Xishuangbanna, Southwest China.** (a) Rainfall and stream water temperature dynamic during 2009 and 2010. (b) Sampling date and the runoff dynamic during 2009 and 2010.
(TIF)

Figure S2 **Dynamics of different carbon components of sampling time during 2009–2010.** (a) Dynamics in concentration of TIC, DIC, and PIC. (b) Dynamics in concentration of TOC, DOC, and POC.
(TIF)
