# Supplementary figures and images for: The Role of Stream Water Carbon Dynamics and Export in the Carbon Balance of a Tropical Seasonal Rainforest, Southwest China
Source: PLoS One. 2013 Feb 20;8(2):e56646. doi: 10.1371/journal.pone.0056646 (PMC3577870; doi:10.1371/journal.pone.0056646)

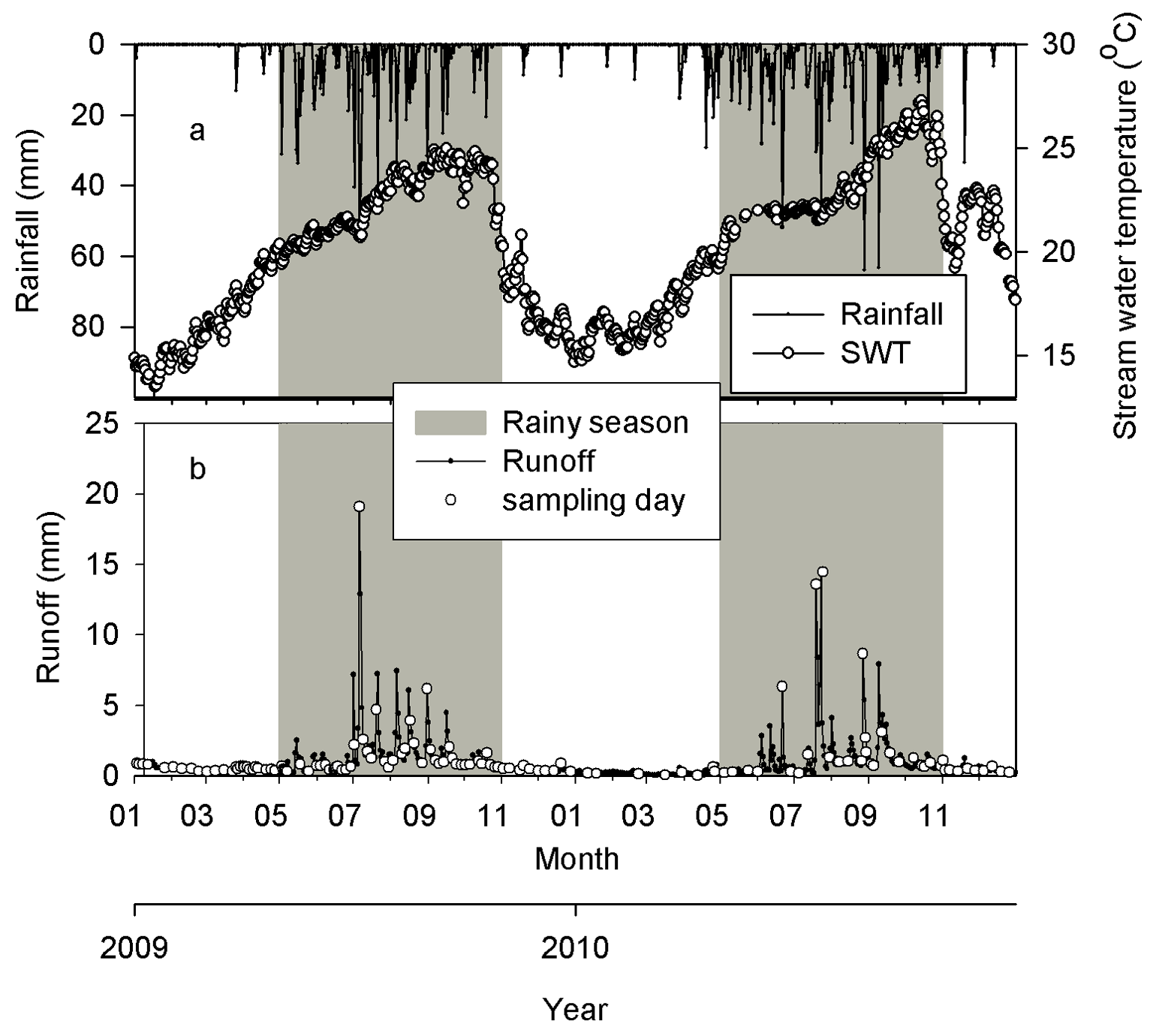

Supplement: Figure S1 — Sampling date of stream water 2009–2010 in tropical seasonal rainforest at Xishuangbanna, Southwest China. (a) Rainfall and stream water temperature dynamic during 2009 and 2010. (b) Sampling date and the runoff dynamic during 2009 and 2010. (TIF) [file pone.0056646.s001.tif]

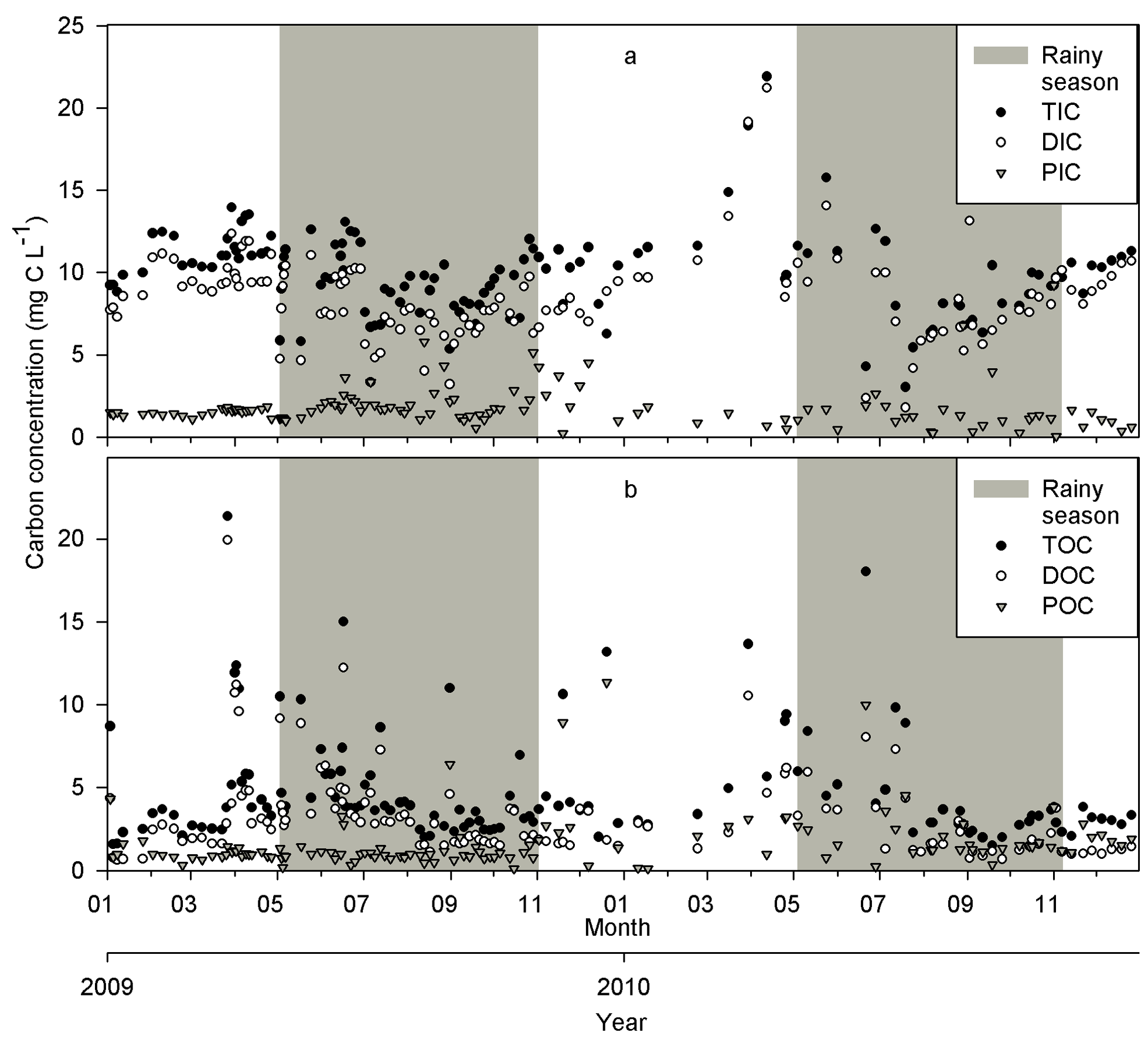

Supplement: Figure S2 — Dynamics of different carbon components of sampling time during 2009–2010. (a) Dynamics in concentration of TIC, DIC, and PIC. (b) Dynamics in concentration of TOC, DOC, and POC. (TIF) [file pone.0056646.s002.tif]
